# Concurrent Lymphoma and COVID-19: Diagnostic and Therapeutic Challenges of High-Grade Lymphoma and COVID-19

**DOI:** 10.7759/cureus.22635

**Published:** 2022-02-26

**Authors:** Jasmin Hundal, Alexander R Vartanov, Kang Huh, Enrique Ballesteros, Victoria Forbes

**Affiliations:** 1 Internal Medicine, University of Connecticut, Farmington, USA; 2 Internal Medicine, University of Connecticut Health, Farmington, USA; 3 Pathology, University of Connecticut Health, Farmington, USA; 4 Hematology-Oncology, University of Connecticut, Farmington, USA

**Keywords:** high-grade b-cell lymphoma, chemoimmunotherapy, cancer care, lymphoma, covid-19

## Abstract

The coronavirus disease 2019 (COVID-19) global pandemic has put an unprecedented strain on cancer care. The initial months were marred by fears of immunocompromised patients becoming opportunistic hosts to this deadly virus. We present a case of newly diagnosed high-grade B-cell lymphoma in a patient with COVID-19 and discuss the diagnostic and therapeutic challenges posed.

A 76-year-old female presented with one month of progressive malaise, poor appetite, weight loss, and night sweats. A surveillance COVID-19 polymerase chain reaction (PCR) resulted positive. With strict isolation precautions, the daily focused physical examination masked several key findings including multifocal adenopathy. She developed hypoxic respiratory failure and progressive transaminitis and cytopenias. Image-guided, rather than excisional, biopsy revealed high-grade B-cell lymphoma. Superimposed COVID-19 infection presented multiple challenges, but she completed treatment and achieved remission. Suspicion for underlying malignancy was high. Institutional concerns included obtaining imaging studies and the gold standard excisional tissue biopsy while maintaining acceptable staff exposure. Fortunately, a lymph node core biopsy confirmed the histopathological diagnosis of high-grade B-cell lymphoma. The administration of chemoimmunotherapy (rituximab, cyclophosphamide, doxorubicin, dose-reduced vincristine, and prednisone (R-CHOP)) posed inherent risks, notably, worsening cytopenias and hepatotoxicity. The approach to treatment was further complicated as the interaction of high-grade lymphoma and COVID-19 remained unclear.

Medical teams have faced delays executing formerly routine diagnostic studies and formulating timely and appropriate treatment strategies. Careful consideration of risks and benefits must be weighed. A multidisciplinary approach is crucial to successfully treat patients. The relationship between COVID-19 and cancer treatment is yet to be established, and large sample-size studies are required.

## Introduction

The coronavirus disease 2019 (COVID-19) global pandemic has put an unprecedented strain on cancer care. Most directly, the initial months were marred by fears of immunocompromised patients becoming opportunistic hosts to this deadly virus. Non-emergent treatments were postponed, and it has been well reported that preventative cancer screening significantly regressed in the United States in 2020 [1]. A year-to-year survey of Medicare claims demonstrated a staggering 70% decrease in new patient evaluation and management visits at the height of the pandemic in April 2020 [1].

Several groups have reported on outcomes of patients with known hematologic malignancies with superimposed COVID-19 infection [2,3]. Notably, a series of 32 patients in the United Kingdom reported a 28-day case mortality rate of 56%, with 89% of deaths attributed directly to COVID-19 [2].

Medical teams have faced delays executing previously routine diagnostic studies and formulating timely and appropriate treatment strategies. We present a case of a newly diagnosed high-grade B-cell lymphoma on a background of COVID-19 positivity. We discuss diagnostic and therapeutic challenges that this case posed and provide a framework for multidisciplinary teams treating patients with newly diagnosed high-grade lymphoma in the COVID-19 era.

## Case presentation

Our patient is a 76-year-old woman in excellent health. Her medication list included metoprolol succinate for sinus tachycardia and multiple as-needed medications for insomnia. She underwent outpatient evaluation of fatigue and intermittent nonspecific gastrointestinal symptoms when her primary care provider identified a marked drop in hemoglobin and recommended an emergency room visit for further investigation.

She endorsed one month of progressive fatigue, malaise, poor appetite, weight loss, and night sweats. She stated that she was becoming more dyspneic with minimal exertion limiting her work in her garden.

The patient was febrile to 101.6°F but otherwise hemodynamically stable on arrival. Initial laboratory findings revealed the following: white blood cell count of 9,900/uL, hemoglobin of 9.5 g/dL, hematocrit of 28.3%, platelet count of 71,103/uL, 85% neutrophils, 10% bands, and 4% monocytes. Liver function tests demonstrated an aspartate aminotransferase (AST) level of 97 U/L, alanine aminotransferase (ALT) level of 61 U/L, and alkaline phosphatase level of 158 U/L. There was initial concern for tick-borne illnesses given her constellation of symptoms, frequent outdoor activity, cytopenias with transaminitis, and residence in New England. Serologies were collected. A surveillance COVID-19 polymerase chain reaction (PCR) test, performed in the emergency room per hospital protocol, returned positive. The presenting symptoms and laboratory abnormalities were attributed to COVID-19 infection.

**Figure 1:**
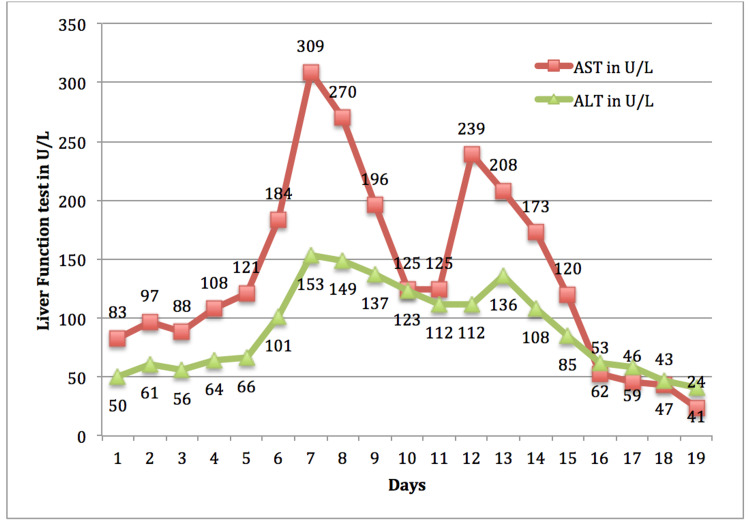
The trend of AST and ALT. Initial uptrending values were attributed to COVID-19 infection rather than direct hepatic injury from lymphoma, and dose-adjusted R-CHOP was started on days 10 and 11. AST, aspartate aminotransferase; ALT, alanine aminotransferase; R-CHOP, rituximab, cyclophosphamide, doxorubicin, dose-reduced vincristine, and prednisone.

With strict isolation precautions, the patient’s daily focused physical examination masked several critical findings, including palpable right supraclavicular, axillary, and inguinal adenopathy. Serial laboratory studies over the initial 48 hours of admission demonstrated worsening cytopenias and progressive hepatic injury despite a stable respiratory status. The platelet count dropped to 57,000/uL. The liver function test trend is shown in Figure 1. Her reticulocyte index was 0.11, suggestive of an underlying hypoproliferative process versus myelosuppression in the setting of acute infection.

**Figure 2:**
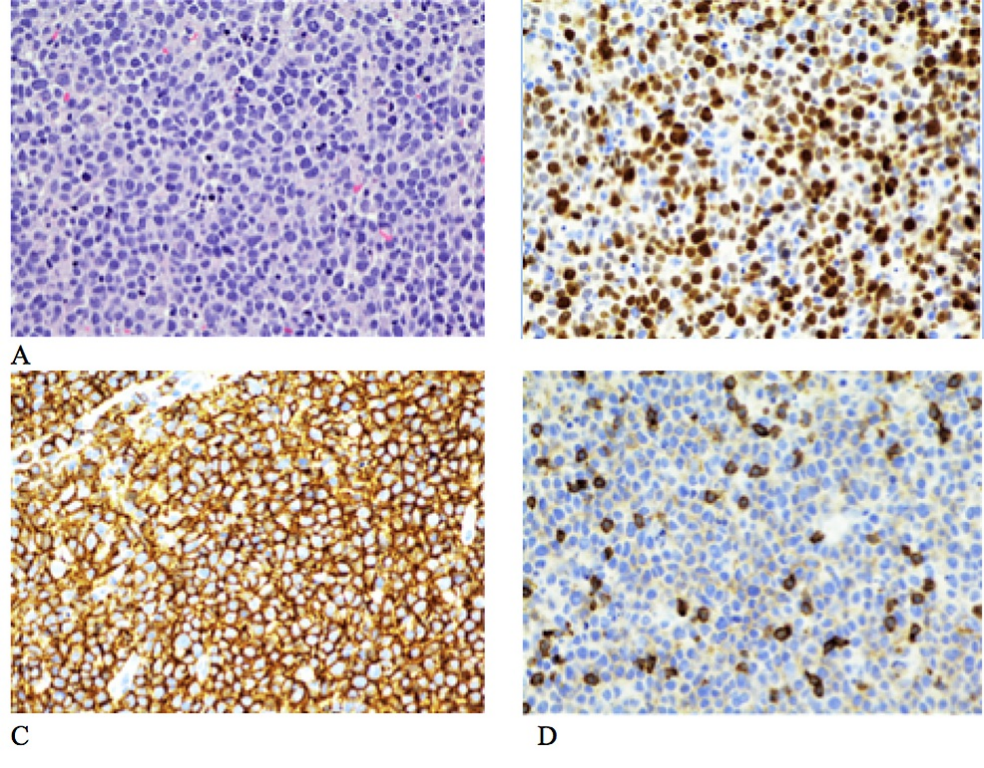
(A) Histopathology of the lymph node biopsy specimen revealed medium- to large-sized neoplastic cells with generally round nuclei (hematoxylin and eosin, 40x). Immunohistochemistry of the lymph node biopsy specimen revealed CD20 and M1B1 expression (B-C) and weak expression of CD5 (D). MYC expression was negative by fluorescence in situ hybridization (FISH) (not shown).

A CT scan of the abdomen and pelvis demonstrated marked splenomegaly of 19 cm with ill-defined hypoechoic regions of the liver and extensive mediastinal, hilar, axillary, inguinal, pelvic, and retroperitoneal lymphadenopathy, with nodes measuring 2.4 cm in the right inguinal region and a 4 cm conglomerate of mesenteric nodes in the mid-abdomen. She developed mild respiratory symptoms necessitating supplemental oxygen by nasal cannula. Although an excisional lymph node biopsy was indicated for precise pathological subclassification and diagnosis of a lymphoproliferative disorder, the inherited risk of elective endotracheal intubation and operative exposure to aerosolized severe acute respiratory syndrome coronavirus 2 (SARS-CoV-2) virus posed a diagnostic challenge. After careful consideration and exhaustive interdisciplinary discussion, we pursued an image-guided needle biopsy. Tissue yield was sufficient to diagnose high-grade B-cell lymphoma, favoring diffuse large B-cell lymphoma (DLBCL), not otherwise specified (NOS), by 2016 World Health Organization criteria. Specifically, the morphology of medium- to large-sized pleomorphic cells with prominent nucleoli was alone, and not diagnostic of DLBCL. Still, in the presence of immunohistochemistry profile showing co-expression of CD19, CD20, kappa, as well as CD5, CD2, and CD79a with a Ki-67 of 90%, this diagnosis was favored (Figure 2). Moreover, fluorescence in situ hybridization (FISH) did not reveal rearrangements of B-cell lymphoma 6 (BCL6), MYC, MYC/IGH, or IGH/B-cell lymphoma 2 (BCL-2), guiding treatment decisions. The patient was started on rituximab, cyclophosphamide, doxorubicin, dose-reduced vincristine, and prednisone (R-CHOP).

Her immediate post-biopsy course was complicated by persistent hypoxic respiratory failure and progressive transaminitis secondary to COVID-19 infection. The patient was illness 10 for her COVID-19 disease. She received prednisone as an alternative to 6 mg of dexamethasone but was transitioned to dexamethasone after chemotherapy. The patient did not receive remdesivir given hepatic and renal failure and initiation of chemotherapy. For COVID-19 treatment, she received one dose of convalescent plasma and supplemental oxygen.

An MRI demonstrated dural enhancement and she received intrathecal methotrexate. Acyclovir was started for prophylaxis. After the first cycle, the patient developed febrile neutropenia and received vancomycin and cefepime. After discussing with the infectious disease team regarding the possible adverse effects of granulocyte colony-stimulating factor (G-CSF) in the setting of COVID-19, the patient was started on filgrastim. She received nine days of growth factor, 12 units of irradiated platelets, and leukocyte-reduced packed red blood cells. Her respiratory status gradually improved, cytopenias recovered, and she was discharged home following cycle one of chemoimmunotherapy (CIT). She followed up with her hematologist/oncologist and completed six cycles of R-CHOP with six cycles of intrathecal methotrexate.

## Discussion

This case identified multiple challenges in delivering inpatient cancer care in the COVID-19 era. Institutional considerations included obtaining imaging studies and tissue biopsies while maintaining acceptable staff exposure. The suspicion for underlying lymphoma was high in our patient’s case, and the first challenge was to obtain the gold standard diagnostic excisional biopsy of the lymph node. Interventional radiology studies demonstrate comparable diagnostic accuracy of needle core biopsies with excisional biopsy [4]. To minimize aerosol-generating procedures and exposure, an image-guided needle biopsy was pursued.

Subsequently, with the histopathological diagnosis of a high-grade B-cell lymphoma, NOS, the administration of CIT posed inherent risks, most notably, profound cytopenias and hepatotoxicity. Rituximab is a monoclonal antibody directed against the CD20 surface antigen on B-lymphocytes, leading to depletion of lymphocytes, which are vital components of a robust immune system [5]. A common finding with lymphoma and COVID-19 infection is lymphopenia. Intensive chemotherapy causes severe myelosuppression and increases the risk of developing superimposed infection with SARS-CoV-2 infection. Interestingly, data from two studies indicated a favorable, beneficial relationship between lymphopenia and the severity of COVID-19 infection in patients with underlying hematological malignancies [6,7]. Despite exhibiting cytopenias, this patient also successfully underwent CIT and COVID-19 treatment without significant complications. However, data are limited, and further investigations are required to determine the implication of lymphopenia in COVID-19 patients with hematologic malignancies.

The approach to treatment was further complicated as the interaction of high-grade lymphoma and COVID-19 remained unclear. The initial administration of R-CHOP was complicated by multiple oxygen desaturation events requiring administration of supplemental oxygen by nasal cannula. As shown in Figure 1, the uptrending liver enzymes predated a lymphoma diagnosis. Chemotherapy was warranted, but hepatic dose adjustments were considered. Did the elevated liver enzymes represent hepatic infiltration by lymphoma or changes secondary to COVID-19? It was unclear whether the pathogenesis of COVID-19 versus lymphoma could explain these findings. Transaminitis appeared to stabilize with CIT. Our multidisciplinary discussion attributed the inflammation to COVID-19 and determined that the patient would benefit from dose adjustments of vincristine.

Our patient demonstrated severe cytopenias and received filgrastim, a G-CSF, to minimize the complications of neutropenia. It is a well-tolerated, routine, and safe medication. G-CSF induces both differentiation and proliferation of myeloid progenitors, stimulates the production of granulocytes and macrophages, and promotes the survival of target cells; however, an imbalance in this production and signaling can lead to harmful effects in the setting of acute inflammatory conditions [8]. National guidelines recommend the administration of G-CSF to minimize neutropenia and decrease the risk of predisposing patients to SARS-CoV-2 or developing complications of neutropenia. However, the safety of administering G-CSF in acutely ill patients with confirmed COVID-19 is unknown. COVID-19 causes a severe inflammatory state and leads to the stimulation of pro-inflammatory cytokines. Administration of the potent stimulating growth factor can lead to a further heightened inflammatory state. A rapid rise in neutrophils can cause alveolar hemorrhage [8]. Two reports have demonstrated a rapid clinical decline and development of severe respiratory disease after G-CSF administration [8]. However, our case demonstrates successful administration of filgrastim in a patient with acute COVID-19 infection and active chemotherapy. Larger, randomized control clinical studies are required to investigate G-CSF administration in COVID-19 patients.

The careful consideration of risks and benefits of targeted treatment must be weighed in the face of increased mortality risk with chemotherapy-associated immunosuppression in patients co-infected with SARS-CoV-2 [9,10]. A retrospective, multicenter cohort study demonstrated poor prognoses in patients with hematological malignancies who received chemotherapy within four weeks before symptom onset of COVID-19 [10]. Lower respiratory tract infections from COVID-19 in patients with hematological malignancies have been associated with high rates of oxygen dependence and mortality compared to solid tumors [11]. A two-fold increased risk of death with a 28-day mortality of 39% and a four-fold risk of undergoing intensive treatment was reported [7]. In contrast, additional retrospective studies revealed that chemotherapy does not significantly increase mortality in patients with COVID-19 compared with patients with cancer who have not received chemotherapy [12,13].

## Conclusions

The COVID-19 global pandemic has put an unprecedented strain on cancer care. Medical teams have faced delays executing formerly routine diagnostic studies and formulating timely and appropriate treatment strategies. We presented a case of a newly diagnosed high-grade B-cell lymphoma in a patient with COVID-19 and discussed the diagnostic and therapeutic challenges posed. Institutional concerns included obtaining imaging studies and the gold standard excisional tissue biopsy while maintaining acceptable staff exposure. Furthermore, the administration of CIT (R-CHOP) posed inherent risks, notably, worsening cytopenias and hepatotoxicity. The approach to treatment was further complicated as the interaction of high-grade lymphoma and COVID-19 remained unclear. This case highlights the importance of a multidisciplinary approach to treating patients successfully and careful consideration of risks and benefits must be weighed. The relationship between COVID-19 and cancer treatment is yet to be established. Unfortunately, a clear relationship between COVID-19 infection and cancer has yet to be found and large sample-sized studies are needed.
